# Evaluation of Alternative Methods to Assess the Biological Properties of Propolis on Metabolic Activity and Biofilm Formation in *Streptococcus mutans*

**DOI:** 10.1155/2019/1524195

**Published:** 2019-08-18

**Authors:** Jorge Jesús Veloz, Marysol Alvear, Luis A. Salazar

**Affiliations:** ^1^Departamento de Ciencias Biológicas y Químicas, Facultad de Medicina y Ciencia, Universidad San Sebastián, Campus Los Leones, Lota 2465, 7510157 Providencia, Santiago, Chile; ^2^Departamento de Ciencias Químicas y Recursos Naturales, Facultad de Ingeniería y Ciencias, Universidad de La Frontera, Avenida Francisco Salazar 01145, 4811230 Temuco, Chile; ^3^Center of Molecular Biology and Pharmacogenetics, Scientific and Technological Bioresource Nucleus (BIOREN), Universidad de La Frontera, Avenida Francisco Salazar 01145, 4811230 Temuco, Chile

## Abstract

Several biological activities have been reported for the Chilean propolis, among their antimicrobial and antibiofilm properties, due to its high polyphenol content. In this study, we evaluate alternative methods to assess the effect of Chilean propolis on biofilm formation and metabolic activity of *Streptococcus mutans* (*S. mutans*), a major cariogenic agent in oral cavity. Biofilm formation was studied by using crystal violet and by confocal microscopy. The metabolic activity of biofilm was evaluated by MTT and by flow cytometry analysis. The results show that propolis reduces biofilm formation and biofilm metabolic activity in *S. mutans*. When the variability of the methods to measure biofilm formation was compared, the coefficient of variation (CV) fluctuated between 12.8 and 23.1% when using crystal violet methodology. On the other hand, the CV ranged between 2.2 and 3.3% with confocal microscopy analysis. The CV for biofilm's metabolic activity measured by MTT methodology ranged between 5.0 and 11.6%, in comparison with 1.9 to 3.2% when flow cytometry analysis was used. Besides, it is possible to conclude that the methods based on colored compounds presented lower precision to study the effect of propolis on biofilm properties. Therefore, we recommend the use of flow cytometry and confocal microscopy in *S. mutans* biofilm analysis.

## 1. Introduction

Propolis is a product collected by honeybees (*Apis mellifera*) and formed by the resinous excretions of buds and bark of trees and shrubs [1]. Propolis is widely used for its medicinal properties. Several biological activities have been reported for propolis including anti-inflammatory, antibacterial, antifungal and/or antiviral, immunomodulatory properties, suppression of HIV-1 replication and immunoregulatory effect, cytotoxicity, hepatoprotection, and free-radical scavenging activity [2–5].

Similarly, the Chilean propolis has showed different biological properties, including antiangiogenic [6–8], antiatherosclerotic [7, 9], antifungal [10], antidiabetic [11], antimicrobial [12–16], hepatoprotective [17], antiproliferative [18], and antioxidant [18] activities, due to its high content of polyphenols, mainly pinocembrin [13].

In relation to antimicrobial activity, our group has demonstrated specifically the antibiofilm properties of the Chilean propolis against *Streptococcus mutans* (*S. mutans*) [14–16], a major cariogenic agent in oral cavity. Biofilms are clusters of single or multiple species of bacteria encased in a matrix composed of polysaccharides, proteins, and DNA that protect the bacteria from environmental pressures. There are many protocols that have been proposed to analyze relative biofilm formation [19]. However, some assays cannot usually distinguish between planktonic killing by the antibiotic and specific antibiofilm effects since bacteria are exposed to the compound of interest before they have a chance to adhere [20]. Similarly, assaying residual bound bacteria using crystal violet, the most widely used method to evaluate biofilm activity, has issues since crystal violet stains biomass rather than living bacteria, and thus, dead bound bacteria will still be stained [19, 20]. Thus, it is important to assess the best method to monitor and to analyze biofilm growth in the presence of antibiofilm/antimicrobial agents. Here, we compared various methods for quantifying the antibiofilm activity of Chilean propolis on *S. mutans*, including crystal violet staining, metabolic dyes, flow cytometry, and confocal microscopy.

## 2. Materials and Methods

### 2.1. Preparation of Polyphenol-Rich Extract of Propolis (EP)

To evaluate the effect of polyphenols from EP in antimicrobial activity and biofilm formation of *S. mutans*, propolis was collected during the Spring of 2008 from the La Araucanía region (Chile). A crude propolis sample was kept frozen (−20°C) and later crushed in cold, and 30 grams was dissolved in 100 mL of ethanol (70%) and macerated for 7 days at room temperature. The ethanolic extract of propolis (EEP) was filtered with a Whatman 2.0 paper and centrifuged at 327 g, during 20 minutes at 5°C. Finally, the solvent was evaporated at a temperature of 40°C, for 2 hours in a rotavaporator (Buchi, R-210, Germany) and dissolved for 24 h with sterile DMSO (0.01%) to obtain polyphenol-rich extract of propolis (EP).

### 2.2. Determination of Total Phenolic Content in EP

The content of total polyphenols in EP was quantified by Folin–Ciocalteu reaction by a modification of Popova and collaborator's methodology [21]. For this assay, 100 *μ*L of EP was mixed with 100 *μ*L of distilled water and 2 mL of Folin–Ciocalteu reagent (Merck, Germany). The resulting solution was incubated for 8 minutes, and finally, 3 mL of sodium carbonate (20%) (w/v) was added. The absorbance of this solution was measured at 760 nm after 2 hours of incubation at room temperature. The concentration of polyphenols was calculated from a calibration curve and was expressed in mg·mL^−1^ equivalent to the pinocembrin-galangin standard mixture (1 : 1).

### 2.3. Bacteria Culture Conditions and Inoculum

Bacteria were obtained from clinical isolates from children with tooth decay. *S. mutans* was identified using the methodology proposed by Salazar et al. [22]. The cultures were made in Petri plates with Columbia agar (Becton Dickinson and Co., NY, and USA) supplied with sucrose (1%) in an anaerobic container (GasPak EZ. Becton Dickinson and Co., NY, USA) and it was incubated at 37°C and 5% of CO_2_, for 24 hours. The inoculum was adjusted using optical density comparison from 1.0 to 550 nm which corresponds to 2 × 10^8^ CFU·mL^−1^ (stock suspension).

### 2.4. Determination of Antibacterial Activity

Minimum inhibitory concentration (MIC) was determined by the serial dilution method following the NCCLS guidelines [23]. The suspension of 5 × 10^5^ CFU·mL^−1^ was inoculated in 96-well microplates, containing 100 *μ*L of sterile trypticase soy broth (Becton Dickinson and Co., NY, USA) with sucrose 1% and with different concentrations of polyphenol-rich extract of propolis (EP) (from 0.1 to 1.96 *μ*g·mL^−1^). Chlorhexidine digluconate (0.2%) was used as positive control, and cultures in DMSO (0.01%) without propolis were used as a negative control. The assay was performed in triplicate and incubated for 48 hours.

### 2.5. Biofilm Formation and *S. mutans* Adherence

Biofilm growth was quantified and indirectly assessed by crystal violet staining assay. The *S. mutans* attachment cells were grown in microplates with sterile trypticase soy broth (TSB) and sucrose (1%). *S. mutans* cultures were supplied with concentrations of EP, between 0.1 and 1.96 *μ*g·mL^−1^, under anaerobic conditions 37°C and 5% of CO_2_, for 48 hours. First, the broth was removed, the plates were washed three times to eliminate nonadherent bacteria with PBS, and the plates were dried at 60°C for 45 minutes. After that, each well was stained with 100 *μ*L of crystal violet 1% (w/v) solution, incubated for 15 minutes, and washed again with sterile PBS. Biofilm formation was determined by adding 125 *μ*L of ethanol 95% per well and then transferred to a new plate to measure the optical density (OD) at 590 nm in a microplate reader in comparison with the control biofilm (without EP) [24].

### 2.6. Biofilm Growth for Morphology Analysis

For biofilm generation, samples were prepared in FluoroDish plates (World Precision Instrument Inc., China) that contained 3 mL of sterile tripticase soy broth supplied with sucrose, 1%. The plates were prepared with 10 *μ*L of bacterial inoculum (5 × 10^5^ UFC·mL^−1^) for incubation at 5% of atmosphere CO_2_, at 37°C for 48 hours. Biofilm was generated in a liquid medium, and different concentrations of EP were added (0.1 to 1.96 *μ*g·mL^−1^).

### 2.7. Fluorescent Labeling

Biofilms were stained with calcein Biofilm Tracer™ (Invitrogen, the USA). First, the plates were incubated with 50 *μ*L of calcein probe and were incubated for 1 hour. After incubation, a medium was removed and, then, the plates were washed three times using PBS to eliminate unabsorbed tracer [22].

### 2.8. Microscopic Analysis of Biofilm Thickness

A 60 × 0.21 NA objective lens was used to visualize bacterial plaque by means of the confocal scanning laser microscope Olympus Fluoview 100. For imaging antimicrobial effect, the 480 nm laser was used for excitation and the ﬂuorescent signal was detected in a green channel. All images were captured by directed acquisition by *Z*-step, ranging to take a series of time-lapse image scans (512 × 512 pixels) at intervals of 15 seconds and 0.5 *μ*m for each confocal plane. Data were analyzed by ImageJ Mac Biophotonic software.

### 2.9. Analyses of Biofilm Metabolic Activity in 96-Well Microplates

EP cytotoxicity was performed by the modified reduction assay of 3-[4,5-dimethylthiazol-2yl]-2,5-diphenyltetrazolium (MTT) bromide (Sigma-Aldrich, France). Because of this, the reagent was dissolved in PBS, taking it to a concentration of 5 mg·mL^−1^, and added in microplate wells containing 90 *μ*L of trypticase soy broth, plus sucrose (1%). Besides, 10 *μ*L of a bacterial suspension equivalent to 5 × 10^4^ CFU·mL^−1^ was added and supplied with concentrations of EP (0.1 to 1.6 *μ*g·mL^−1^) and 200 *μ*L of MTT to obtain formazan. The plates were incubated at 37°C and 5% CO_2_ for 48 hours. The absorbance of obtained solutions was quantified at 490 nm. Cytotoxicity was expressed in percentages of inhibition in the cellular viability in cultures with less than 50% growth (IC50%) and compared to the color developed by untreated cells. The controls included DMSO at 0.01% as vehicle control, and untreated cells (negative control) and chlorhexidine digluconate at 0.2% as positive control. All the tests were carried out in triplicate.

The inhibition percentage of biofilm viability was calculated using the following formula:

(1) $$\begin{array}{c} \text{inhibition percentage}=\frac{\text{control OD}570-\text{treated OD}570}{\text{control OD}570}*100. \end{array}$$

### 2.10. Biofilm Viability by Flow Cytometry

A biofilm was formed in glass tubes at anaerobial conditions at 37°C and for 48 hours using sterile trypticase soy broth (TSB), sucrose (1%), and EP (0.1 to 1.96 *μ*g·mL^−1^) to a final volume of 10.000 *μ*L. After incubation, an aliquot of 100 *μ*L of biofilm suspension was transferred to an eppendorf and was stained with Live/Dead® BacLight Bacterial Viability Kit. First, 10 *μ*L of Syto9 (20 *μ*M) was added for 15 minutes, and later, 10 *μ*L of PI (800 *μ*M) was added for 5 minutes; the tubes were washed twice with sterile PBS and centrifuged for 1 minute at 5000*g*. Finally, cells were resuspended in sterile PBS, and they were analyzed by flow cytometry (FACS CantoII™, BD Biosytem). The sample with two stain components was excited at 488 nm, and the emission was registered using the FITC channel for Syto 9 (530/30) and PerCP channel (670/LP) for propidium iodide. Suspension containing 5 × 10^6^ cell·mL^−1^ were aspirated with a flow rate 12 *μ*L·min^−1^. The results of biofilm cell viability were expressed in percentage in relation with untreated control cells [24].

### 2.11. Statistical Analysis

Statistical analyses were performed using the computational Statistical Program R, version 3.5.1. The D'Shapiro–Wilson test was applied to determine the results' normal distribution. Afterwards, the values were analyzed using the nonparametric Wilcoxon test for related samples. The values are expressed as median ± SD. The variability of the evaluated methods was determined by calculating the coefficient of variation (CV). Significant differences were considered at *p* < 0.05.

## 3. Results

### 3.1. Total Polyphenol Content in Chilean Propolis

The content of polyphenols in EP in equivalence of the pinocembrin-galangin mixture was quantified by Folin–Ciocalteu reaction, and it was 137.7 ± 0.7 mg·g^−1^. Previous studies of our group described the chemical composition of the EP. The main flavonoids identified in the Chilean propolis by means of the HPLC-DAD as quercetin, apigenin, pinocembrin, and caffeic acid phenethyl ester (CAPE) [13, 16].

### 3.2. Biofilm Inhibition Assessed by Crystal Violet Staining

**Figure 1:**
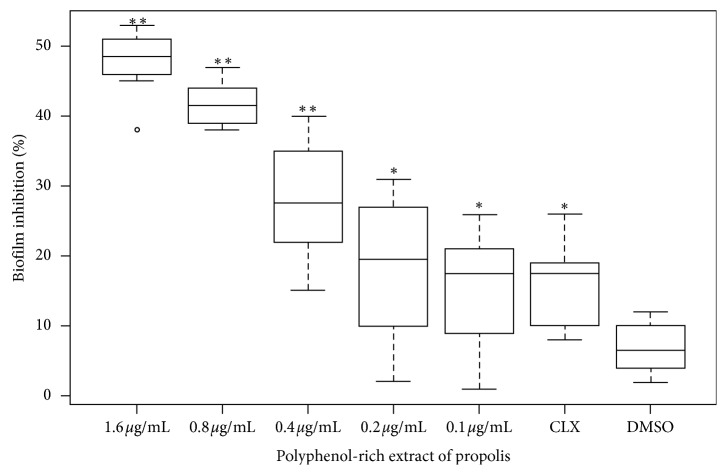
Biofilm inhibition evaluated by crystal violet staining. *S. mutans* cultures were treated with different polyphenol-rich extract of propolis concentrations (0.1 to 1.6 *μ*g·mL^−1^). CLX, chlorhexidine gluconate 0.2% (positive control); DMSO (vehicle control). ^*∗*^*p* < 0.05 or ^*∗∗*^*p* < 0.01 from the nonparametric Wilcoxon test when compared to untreated cells (100% of growth biofilm). Values are expressed as median ± SD.

The percentage of biofilm inhibition in cultures was calculated considering untreated cells as 100% of biofilm growth (control). When EP was added at 1.96 *μ*g·mL^−1^ and 0.8 *μ*g·mL^−1^, the values of biofilm reduction were 53.4 ± 2.0% and 47.7 ± 2.0%, respectively (*p* < 0.01). Similarly, when we used EP at a concentration of 0.4 *μ*g·mL^−1^, the bacterial pellicle decreased in 43.5 ± 3.3% (*p* < 0.01). Also, when we used EP at 0.2 and 0.1 *μ*g·mL^−1^, these concentrations prevented the biofilm growth in 24.9 ± 4.0% and 19.2 ± 3.3%, respectively (*p* < 0.05). Finally, chlorhexidine and DMSO reduced the biofilm formation in 18.8 ± 2.9% and 8.8 ± 2.1%, respectively (Figure 1).

### 3.3. Biofilm Formation Analysis by Confocal Microscopy

**Figure 2:**
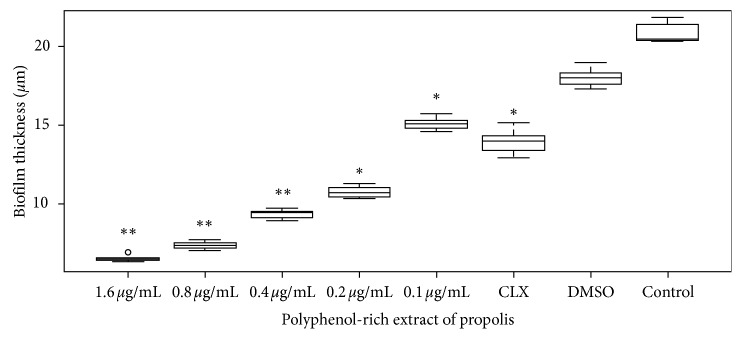
Effect of different polyphenol-rich extract of propolis concentrations in biofilm formation by confocal microscopy. Antibiofilm activity was expressed as thickness (median ± SD) in *S. mutans* cultures treated with different polyphenol-rich extract of propolis concentrations. ^*∗*^*p* < 0.05 and ^*∗∗*^*p* < 0.01 from the nonparametric Wilcoxon test when compared to untreated cells.

The extracellular matrix (biofilm) obtained from *S. mutans* cultures treated with EP at a concentration of 0.8 *μ*g·mL^−1^ shows a size of 7.3 ± 0.2 *μ*m in comparison to the control biofilm (20.8 ± 0.3 *μ*m); when 0.4 *μ*g·mL^−1^ of polyphenols was applied, the obtained biofilm was 9.3 ± 0.2 *μ*m; other concentrations such as 0.2 *μ*g·mL^−1^ and 0.1 *μ*g·mL^−1^ generated greater biofilms, with 10.7 ± 0.2 *μ*m and 15.0 ± 0.2 *μ*m. Although the effect of chlorhexidine was higher than these low concentrations, the synthetic compound allowed sizes of 13.9 ± 0.6 *μ*m. This may probably be because excipients contained in their formulation may be affecting this result.  Figure 2 shows the effect of different polyphenol concentrations in biofilm thickness.

### 3.4. Analyses of Biofilm's Metabolic Activity by MTT

**Figure 3:**
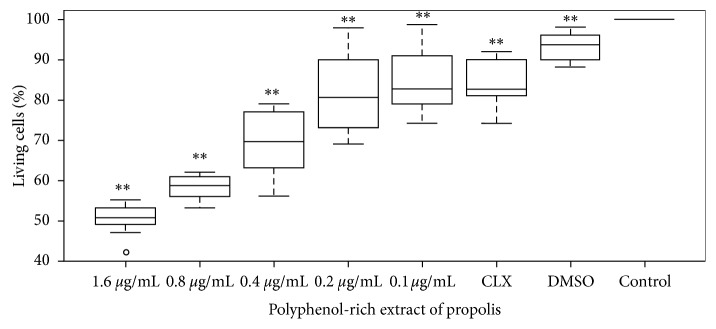
Biofilm metabolic activity determined by the MTT method. The effect of polyphenol-rich extract of propolis in *S. mutans* cultures was quantified in 96-well microplates. The percentage of living biofilm cells was expressed as mean ± standard deviation. CLX, chlorhexidine digluconate 0.2% (positive control), DMSO (vehicle control). ^*∗∗*^*p* < 0.01 from the nonparametric Wilcoxon test when compared to untreated cells.

The MTT method showed significant reduction values in a percentage of cellular viability for concentrations lower than MIC. For the EP concentration at 0.8 *μ*g·mL^−1^, the reduction was 58.2 ± 3.5%; for EP at 0.4 *μ*g·mL^−1^ was 69.2 ± 8.6%; for EP at 0.2 *μ*g·mL^−1^ was 82.1 ± 8.8%, and for EP at 0.1 *μ*g·mL^−1^ was 85.0 ± 7.2%. The reduction for chlorhexidine was 83.0 ± 7.0%, with significant statistical differences. Cellular viability at different EP concentrations is shown in Figure 3.

### 3.5. Biofilm Metabolic Activity of *S. mutans* by Flow Cytometry

**Figure 4:**
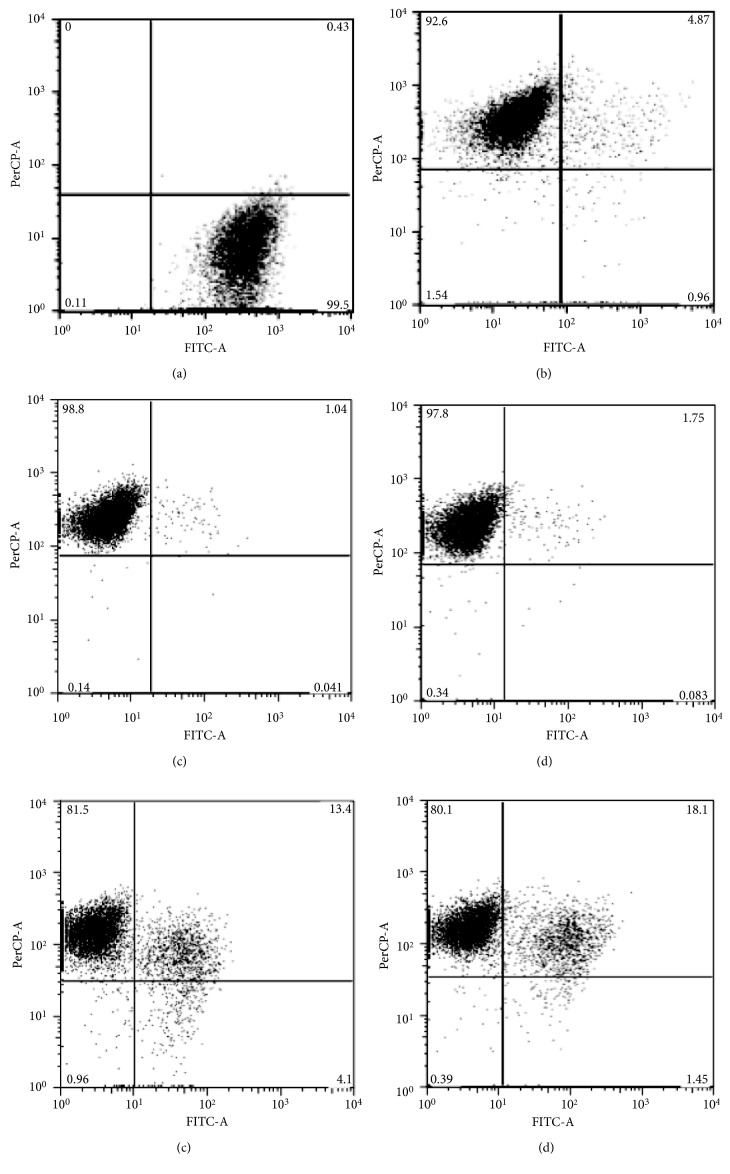
Metabolic activity in *S. mutans* biofilm measured by flow cytometry. The biofilm cellular suspension was excited at 488 nm, and emission was registered with FITC channel for Syto 9 (530/30) and PerCP channel (670/LP) for propidium iodide. (a) Untreated cells. (b) Chlorhexidine. (c) EP at 0.8 *μ*g·mL^−1^. (d) EP at 0.4 *μ*g·mL^−1^. (e) EP at 0.2 *μ*g·mL^−1^. (f) EP at 0.1 *μ*g·mL^−1^. EP, polyphenol-rich extract of propolis.

**Figure 5:**
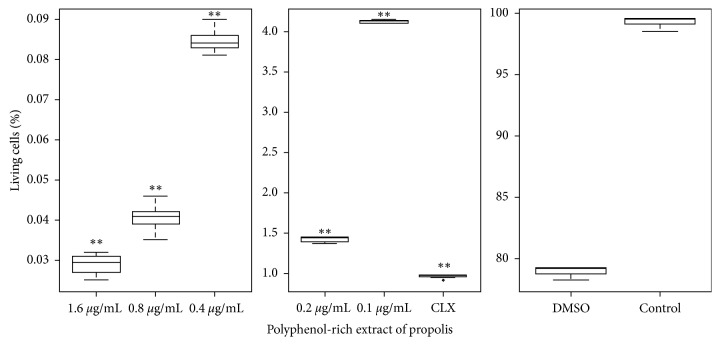
Effect of different concentrations of polyphenol-rich extract of propolis (EP) on biofilm metabolic activity, determined by flow cytometry in *S. mutans*. The percentage of biofilm living cells was expressed as median ± SD. ^*∗∗*^*p* < 0.01 from the nonparametric Wilcoxon test when compared to untreated cells (control).

Figure 4 shows individual dot plots of the *S. mutans* biofilm analyzed by flow cytometry; the assays to measure metabolic activity in the *S. mutans* biofilm generated for 48 hours facilitated the differentiation of live and dead cell populations performed with excitation/emission fluorescence Syto 9 and propidium iodide stains. Some EP concentrations such as 0.8 *μ*g·mL^−1^ (0.04 ± 0.005% of live cells) and 0.4 *μ*g·mL^−1^ (0.08 ± 0.004% of live cells) had a higher effect than chlorexidine in the reduction of live cells detected; the value for chlorexidine was 0.95 ± 0.003%. For the EP at 0.2 *μ*g·mL^−1^ and EP at 0.1 *μ*g·mL^−1^, a low number of detected viable cells but less than chlorhexidine was observed (1.41 ± 0.004 and 4.1 ± 0.008 of live cells; Figure 5). We found higher statistical differences in comparison with untreated cells in all treatments (*p* < 0.01).

## 4. Discussion

The antimicrobial agents in propolis produce changes in biofilm structure and in cellular aggregation due to fluctuations in the levels of protein and enzymatic expression [25]. In this study, we evaluated several methods to assess the antibiofilm activity of the Chilean propolis. When we compared the variability of the methods to measure biofilm formation, the coefficient of variation (CV) fluctuated between 12.8 and 23.1% after using crystal violet methodology. However, the CV ranged between 2.2 and 3.3% when we used confocal microscopy. The high degree of dispersion for crystal violet staining is probably due to the interference by color development. The other factor that explained the differences is that the crystal violet stains biomass rather than living bacteria, and thus, dead bound bacteria will still be stained. In spite of its popularity, crystal violet has certain weaknesses, including nonspecific binding to anionic proteins and other negatively charged molecules, like capsules, lipopolysaccharides, and DNA/nucleic acids, leading to an inability to distinguish between live and dead bacterial populations [19, 20]. These problems contribute to a large variability among samples that may complicate the interpretation of biofilm screening results.

Colorimetric methods such as MTT have been used to quantify metabolic activity in cultures. The basic principle is the conversion by cellular metabolic activity of the substrate into a colored formazan from tetrazolium, later measurable with a spectrophotometer. Our results show that the CV for biofilm's metabolic activity measured by MTT ranged between 5.0 and 11.6%, in comparison with 1.9 to 3.2% when using flow cytometry analysis. The differences between these methodologies can be explained considering that the flow cytometry used propidium iodide costained with Syto9 (LIVE/DEAD staining) as an indicator for cellular membrane integrity. This combination on stable biofilm with calcein probe alone improves the discrimination between live and dead cells [26]. These results confirm that the techniques involving probes or excitation by laser allowed acquire more accurate information.

Nevertheless, it is necessary to establish a gold standard method to validate the accuracy of the evaluated methods. In addition, it is necessary to eliminate interferences that could appear on having used these methodologies, implementing some validation, for example, using culture-based methods as a reference, to assess metabolic activity (MTT methodology) in parallel to DNA staining or minimizing extracellular matrix coharvesting, if harvested cell viability is to be assessed by staining [19].

## 5. Conclusions

These results suggest that the staining methods presented a large variability to evaluate the effect of propolis on biofilm formation and metabolic activity. Flow cytometry and confocal microscopy allowed more accurate results when compared with traditional methodologies. Thus, we recommend the use of flow cytometry and confocal microscopy to evaluate the antimicrobial properties of propolis in *Streptococcus mutans*.

## Data Availability

The data used to support the findings of this study are available from the corresponding author upon request.

## References

[1] Pietta P. G., Gardana C., Pietta A. M. (2002). Analytical methods for quality control of propolis. *Fitoterapia*.

[2] Pasupuleti V. R., Sammugam L., Ramesh N., Gan S. H. (2017). Honey, propolis, and royal jelly: a comprehensive review of their biological actions and health benefits. *Oxidative Medicine and Cellular Longevity*.

[3] Koo H., Rosalen P. L., Cury J. A., Park Y. K., Bowen W. H. (2002). Effects of compounds found in propolis on *Streptococcus mutans* growth and on glucosyltransferase activity. *Antimicrobial Agents and Chemotherapy*.

[4] Libério S. A., Pereira A. L. A., Araújo M. J. A. M. (2009). The potential use of propolis as a cariostatic agent and its actions on mutans group streptococci. *Journal of Ethnopharmacology*.

[5] Netikova L., Bogusch P., Heneberg P. (2013). Czech ethanol-free propolis extract displays inhibitory activity against a broad spectrum of bacterial and fungal pathogens. *Journal of Food Science*.

[6] Cuevas A., Saavedra N., Cavalcate M. F., Salazar L. A., Abdalla D. S. P. (2014). Identification of microRNAs involved in the modulation of pro-angiogenic factors in atherosclerosis by a polyphenol-rich extract from propolis. *Archives of Biochemistry and Biophysics*.

[7] Daleprane J. B., da Silva Freitas V., Pacheco A. (2012). Anti-atherogenic and anti-angiogenic activities of polyphenols from propolis. *The Journal of Nutritional Biochemistry*.

[8] Cuevas A., Saavedra N., Rudnicki M., Abdalla D. S. P., Salazar L. A. (2015). ERK1/2 and HIF1*α* are involved in antiangiogenic effect of polyphenols-enriched fraction from Chilean propolis. *Evidence-Based Complementary and Alternative Medicine*.

[9] Saavedra N., Cuevas A., Cavalcante M. F. (2016). Polyphenols from Chilean propolis and pinocembrim reduce MMP-9 gene expression and activity in activated macrophages. *BioMed Research International*.

[10] Herrera C. L., Alvear M., Barrientos L., Montenegro G., Salazar L. A. (2010). The antifungal effect of six commercial extracts of Chilean propolis on *Candida* spp.. *Ciencia e Investigación Agraria*.

[11] Pacheco A., Daleprane J. B., Freitas V. S. (2011). Effect of Chilean propolis on glucose metabolism in diabetic mice. *International Journal of Morphology*.

[12] Saavedra N., Barrientos L., Herrera C. L., Alvear M., Montenegro G., Salazar L. A. (2011). Effect of Chilean propolis on cariogenic bacteria *Lactobacillus fermentum*. *Ciencia e Investigacion Agraria*.

[13] Barrientos L., Herrera C. L., Montenegro G. (2013). Chemical and botanical characterization of Chilean propolis and biological activity on cariogenic bacteria *Streptococcus mutans* and *Streptococcus sobrinus*. *Brazilian Journal of Microbiology*.

[14] Veloz J. J., Saavedra N., Alvear M., Zambrano T., Barrientos L., Salazar L. A. (2016). Polyphenol-rich extract from propolis reduces the expression and activity of *Streptococcus mutans* glucosyltransferases at subinhibitory concentrations. *BioMed Research International*.

[15] Veloz J. J., Saavedra N., Lillo A., Alvear M., Barrientos L., Salazar L. A. (2015). Antibiofilm activity of Chilean propolis on *Streptococcus mutans* is influenced by the year of collection. *BioMed Research International*.

[16] Veloz J. J., Alvear M., Salazar L. A. (2019). Antimicrobial and antibiofilm activity against *Streptococcus mutans* of individual and mixtures of the main polyphenolic compounds found in Chilean propolis. *BioMed Research International*.

[17] Herrera C. L., Fritz O., Montenegro G., Alvear M., del Sol M., Salazar L. A. (2010). El propóleos reduce la esteatosis hepática inducida por dieta en ratones. *International Journal of Morphology*.

[18] Russo A., Cardile V., Sanchez F., Troncoso N., Vanella A., Garbarino J. A. (2004). Chilean propolis: antioxidant activity and antiproliferative action in human tumor cell lines. *Life Sciences*.

[19] Azeredo J., Azevedo N. F., Briandet R. (2017). Critical review on biofilm methods. *Critical Reviews in Microbiology*.

[20] Haney E., Trimble M., Cheng J., Vallé Q., Hancock R. (2018). Critical assessment of methods to quantify biofilm growth and evaluate antibiofilm activity of host defence peptides. *Biomolecules*.

[21] Popova M., Bankova V., Butovska D. (2004). Validated methods for the quantification of biologically active constituents of poplar-type propolis. *Phytochemical Analysis*.

[22] Salazar L. A., Vásquez C., Almuna A. (2008). Molecular detection of cariogenic streptococci in saliva. *International Journal of Morphology*.

[23] Hecht D. W., Citron D. M., Cox M. (2007). *Methods for Antimicrobial Susceptibility Testing of Anaerobic Bacteria; Approved Standard*.

[24] Kouidhi B., Zmantar T., Bakhrouf A. (2010). Anti-cariogenic and anti-biofilms activity of *Tunisian propolis* extract and its potential protective effect against cancer cells proliferation. *Anaerobe*.

[25] Abranches J., Zeng L., Kajfasz J. K. (2018). Biology of oral streptococci. *Microbiology Spectrum*.

[26] Tawakoli P. N., Al-Ahmad A., Hoth-Hannig W., Hannig M., Hannig C. (2013). Comparison of different live/dead stainings for detection and quantification of adherent microorganisms in the initial oral biofilm. *Clinical Oral Investigations*.

